# Risk Score to Predict 1-Year Mortality after Haemodialysis Initiation in Patients with Stage 5 Chronic Kidney Disease under Predialysis Nephrology Care

**DOI:** 10.1371/journal.pone.0129180

**Published:** 2015-06-09

**Authors:** Toshiki Doi, Suguru Yamamoto, Takatoshi Morinaga, Ken-ei Sada, Noriaki Kurita, Yoshihiro Onishi

**Affiliations:** 1 Department of Nephrology, Hiroshima University Hospital, Hiroshima, Japan; 2 Division of Clinical Nephrology and Rheumatology, Niigata University Graduate School of Medical and Dental Sciences, Niigata, Japan; 3 Department of Nephrology, Anjo Kosei Hospital, Aichi, Japan; 4 Department of Medicine and Clinical Science, Okayama University Graduate School of Medicine, Dentistry and Pharmaceutical Sciences, Okayama, Japan; 5 Department of Innovative Research and Education for Clinicians and Trainees (DiRECT), Fukushima Medical University Hospital, Fukushima, Japan; 6 Department of Healthcare Epidemiology, School of Public Health in the Graduate School of Medicine, Kyoto University, Kyoto, Japan; 7 Institute for Health Outcomes and Process Evaluation Research (iHope International), Kyoto, Japan; The University of Tokyo, JAPAN

## Abstract

**Background:**

Few risk scores are available for predicting mortality in chronic kidney disease (CKD) patients undergoing predialysis nephrology care. Here, we developed a risk score using predialysis nephrology practice data to predict 1-year mortality following the initiation of haemodialysis (HD) for CKD patients.

**Methods:**

This was a multicenter cohort study involving CKD patients who started HD between April 2006 and March 2011 at 21 institutions with nephrology care services. Patients who had not received predialysis nephrology care at an estimated glomerular filtration rate (eGFR) of approximately 10 mL/min per 1.73 m^2^ were excluded. Twenty-nine candidate predictors were selected, and the final model for 1-year mortality was developed via multivariate logistic regression and was internally validated by a bootstrapping technique.

**Results:**

A total of 688 patients were enrolled, and 62 (9.0%) patients died within one year of HD initiation. The following variables were retained in the final model: eGFR, serum albumin, calcium, Charlson Comorbidity Index excluding diabetes and renal disease (modified CCI), performance status (PS), and usage of erythropoiesis-stimulating agent (ESA). Their β-coefficients were transformed into integer scores: three points were assigned to modified CCI≥3 and PS 3–4; two to calcium>8.5 mg/dL, modified CCI 1–2, and no use of ESA; and one to albumin<3.5 g/dL, eGFR>7 mL/min per 1.73 m^2^, and PS 1–2. Predicted 1-year mortality risk was 2.5% (score 0–4), 5.5% (score 5–6), 15.2% (score 7–8), and 28.9% (score 9–12). The area under the receiver operating characteristic curve was 0.83 (95% confidence interval, 0.79–0.89).

**Conclusions:**

We developed a simple 6-item risk score predicting 1-year mortality after the initiation of HD that might help nephrologists make a shared decision with patients and families regarding the initiation of HD.

## Introduction

Although intervention by nephrologists to chronic kidney disease (CKD) patients before initiation of haemodialysis (HD) is increasing, these patients still have poor outcome. Therefore, it is important for the nephrologists to practice shared decision making with CKD patients and their families regarding the initiation of HD based on informed estimates of early mortality risk. Although several risk scores based on the clinical parameters at the initiation of HD have been previously reported, the risk score to predict mortality risk after initiation of dialysis treatment among CKD patients under predialysis nephrology care is scarce.

Couchoud et al. developed a simple clinical score predicting early mortality in CKD patients starting HD [1]. Although this score was a simple integer score, this study only enrolled elderly patients and included those with unplanned initiation of HD. In addition, the estimated glomerular filtration rate (eGFR) at the initiation of HD was not incorporated into this score, despite being a strong prognostic factor [2] that varies by country and should therefore be considered in mortality prediction. For example, the proportion of patients with eGFR>10 mL/min at the initiation of HD was 11% in Japan [3] and 36% in USA [4]. van Diepen et al. developed a predictive model for incident dialysis patients with consideration of residual GFR as a candidate predictor, whereas this model was only applicable to diabetic dialysis patients and difficult to calculate [5]. Therefore, a simple risk score using daily practice data is necessary for nephrologists to predict mortality of CKD patients after initiation of dialysis treatment without restriction of patients’ age or underlying diseases.

Using multicenter clinical data measured immediately before the initiation of HD, we developed and validated a clinical scoring system predicting 1-year survival after initiation of HD for CKD patients.

## Subjects and Methods

### Study Design

We conducted a retrospective cohort study of patients starting HD at 19 tertiary care institutions and two clinics. This study was approved by the ethics committee of the Okayama University Graduate School of Medicine, Dentistry and Pharmaceutical Sciences (authorization number: 538) and registered with the University Hospital Medical Information Network Clinical Trials Registry (UMIN000007862). Informed consent from participants was ruled unnecessary because we retrospectively analyzed data routinely collected from all patients and we analyzed the data anonymously.

### Patients

In this study, renal function was evaluated by eGFR using the following equation developed for Japanese patients: eGFR (mL/min per 1.73 m^2^) = 194 × Serum creatinine^-1.094^ × Age^-0.287^ (× 0.739 if female) [6]. The inclusion criteria for this study were as follows: patients who started HD between April 2006 and March 2011; and eGFR during predialysis nephrology care could be confirmed in medical records and were shown to have reached to 10 mL/min per 1.73 m^2^ to eliminate patients with unplanned HD initiation. The criteria for exclusion from this study were as follows: those whose eGFR could not be confirmed during predialysis nephrology care because of unplanned initiation or failure to measure; those who started HD before their eGFR had reached to 10 mL/min per 1.73 m^2^; withdrawal of HD treatment within one year; and under 20 years old.

### Data Collection and Processing

Data at the initiation of HD were collected from medical records, including demographic information, underlying renal disease, clinical symptoms, laboratory data, Charlson Comorbidity Index (CCI) [7], performance status categorised using scales of the World Health Organization performance status except category 5 (death) [8], and treatment status.

### Outcome Measures & Candidate Predictors

The primary outcome measure was all-cause mortality within one year of starting HD. Candidate predictors were selected according to a literature review and clinical expertise. Candidate predictors need to be biologically plausible and available in the majority of nephrology practice settings. We therefore selected information on 29 variables, including patient demographics (age, gender, primary renal disease [diabetic nephropathy or other renal disease], and body mass index), laboratory data (eGFR, haemoglobin, urea nitrogen, serum potassium, calcium, phosphorus, albumin, and C-reactive protein), CCI, performance status, symptoms related to uremia (fatigue, edema, pulmonary edema, nausea, dysorexia, diarrhea, constipation, other digestive symptoms, hypertension, peripheral neuropathy, psychiatric disorder, haemorrhagic diathesis, diabetic retinopathy, and itch), and nephrologists’ treatments (erythropoiesis-stimulating agent [ESA]) (Table 1). Uremic symptoms are diagnosed by attending physician. In Japan, almost all patients initiating HD hand in the certificate of attending physician for making use of public support. This certificate includes the following items: chest X-ray, fundus feature, peripheral neuropathy, digestive symptom, psychiatric disorder, severe hypertension, and so on. We collected the data about uremic symptoms from these certificate and medical records. We modified CCI by excluding the items related to diabetes and renal disease in this study for the following reasons: all patients have “moderate or severe renal disease”; and diabetes is included in another variable, that is, primary renal disease.

**Table 1:** Candidate predictors and outcome variables.

	Number missing	Analysis cohort (n = 688)	Survived (n = 626)	Died (n = 62)
**Age, year**	0	69 (59–77)	69 (59–76)	73 (65–78)
**Female gender, %**	0	33.4	33.5	32.3
**Body mass index**	23	22.7 (20.6–25.4)	23.0 (20.7–25.5)	21.5 (19.6–23.9)
**Diabetic nephropathy, %**	0	42.6	43.8	30.7
**eGFR, mL/min per 1.73 m** ^**2**^	0	5.43 (4.37–6.71)	5.38 (4.36–6.55)	6.40 (4.89–7.89)
**Urea nitrogen, mg/dL**	2	86.2 (69.5–106.8)	86.1 (70.0–106.0)	90.1 (67.7–116.1)
**Haemoglobin, g/dL**	1	8.7 (7.7–9.7)	8.7 (7.7–9.7)	8.4 (7.4–9.5)
**Serum albumin, g/dL**	34	3.2 (2.8–3.6)	3.3 (2.9–3.6)	3.0 (2.6–3.4)
**Serum potassium, mg/dL**	4	4.5 (4.0–5.0)	4.5 (4.1–5.0)	4.7 (3.7–5.4)
**Serum calcium, mg/dL**	12	7.9 (7.3–8.4)	7.9 (7.3–8.4)	7.8 (7.5–8.6)
**Serum phosphorus, mg/dL**	13	5.7 (4.8–6.7)	5.7 (4.8–6.8)	5.4 (4.8–6.4)
**C-reactive protein, mg/dL**	46	0.21 (0.07–0.99)	0.20 (0.06–0.80)	0.99 (0.18–3.07)
**Modified Charlson Comorbidity Index** ^a^	22			
0, %		45.6	48.9	11.9
1–2, %		42.5	40.7	61.0
≥3, %		11.9	10.4	27.1
**Performance status**	5			
0, %		14.7	15.8	3.2
1, %		40.1	41.9	22.6
2, %		21.2	22.0	12.9
3, %		16.0	13.6	40.3
4, %		7.3	0.6	19.3
**Fatigue, %**	11	73.0	72.5	78.0
**Edema, %**	2	65.5	65.6	63.9
**Pulmonary edema, %**	5	23.9	23.6	26.2
**Nausea, %**	9	36.4	35.6	44.2
**Dysorexia, %**	8	61.9	61.1	70.5
**Diarrhea, %**	7	4.9	4.7	6.6
**Constipation, %**	14	4.9	5.1	3.3
**Other digestive symptom, %**	9	1.3	1.0	5.0
**CNS manifestation, %**	4	3.1	2.4	9.8
**Peripheral nerve abnormalities, %**	8	12.4	12.3	13.1
**Itch, %**	19	10.0	10.5	4.9
**Haemorrhagic diathesis, %**	3	2.3	2.1	4.9
**Hypertension, %**	4	60.7	62.1	45.9
**Diabetic retinopathy, %**	45	35.3	36.1	26.8
**ESA use, %**	6	85.8	87.5	67.7

Continuous variables represented as median with interquartile range in parentheses.

eGFR, estimated glomerular filtration rate; CNS, central nervous system; ESA, erythropoiesis-stimulating agent

^a^Items related to diabetes and renal disease were excluded from the original Charlson Comorbidity Index in the present study.

### Data Description & Handling Missing Data

Descriptive statistics on candidate predictors and the outcome variable are presented as median or percentage as appropriate.

**Fig 1:**
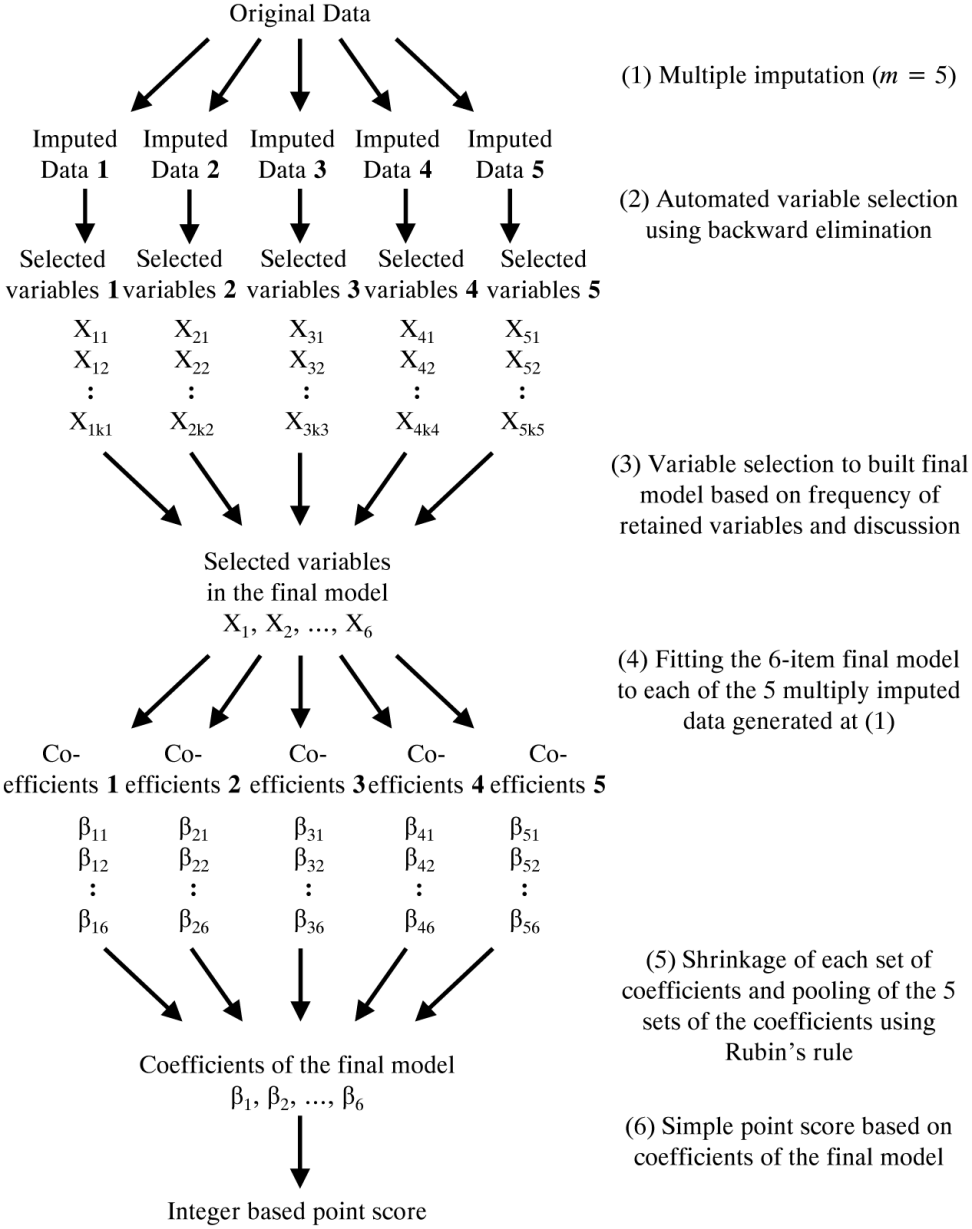
Process of the multiple imputation and derivation of the prediction rule. (1) Five multiply imputed datasets were created using original data. (2) Backward elimination was separately applied to each of the five imputed datasets, resulting in five sets of selected predictors. (3) Predictors that were selected in all of the five data sets were chosen as the final set of selected predictors, with exclusion of some predictors based on balance between number of candidate predictors with number of outcomes (deaths) and discussion according to clinical relevance. (4) The logistic regression with the selected six predictors was separately applied to each of the five imputed data sets, giving five sets of β-coefficients of the six predictors. (5) To avoid overfitting, each of five sets of β-coefficients of the six predictors were shrunken using heuristic shrinkage factor. Then, the mean for each of the five estimates for β-coefficients of the final model were taken and variances of the five estimates were pooled according to Rubin’s rules. (6) The shrunken β-coefficients of the predictors in the final model divided by two-fifths of the two small β-coefficients in the model and rounded up to the nearest integer to give a simple point score.

At least one variable was missing in 183 patients (26.6%). As complete case analysis leads to loss of power and biased results, a multiple imputation approach was utilized to derive predictions for the missing values [9]. Whereas simulation studies have shown that required number of imputations can be as three for data with 20% of missing entries [10], the largest percentage of missing among candidate predictors was 6.7% (46 of 688 patients) for C-reactive protein, and thus five repeated imputations would be considered a conservative choice. Five imputations were performed using multiple imputations with chained equations [10, 11]. Process of the multiple imputation and derivation of the prediction rule was shown as Fig 1.

### Derivation Phase

The association of candidate predictors with 1-year mortality was modeled with multivariate logistic regression. Continuous predictor variables were categorized as appropriate with reference to β-coefficients of bivariate logistic regression models. Selection of predictors was based on backward elimination of variable with P-value>0.157, which is equivalent to the use of the Akaike information criterion (AIC) for model selection [12].

**Fig 2:**
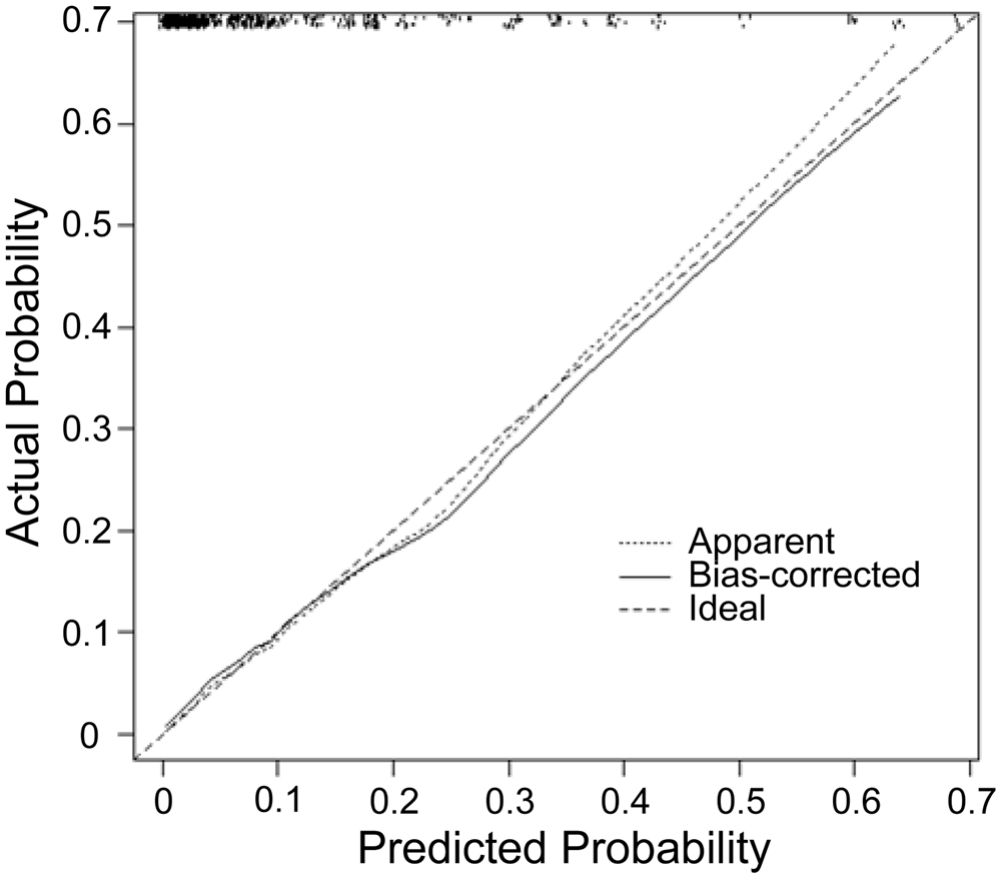
Agreement between the predicted mortality risks and the observed proportions. The short-dashed line (“Apparent”) indicates the agreement between predicted mortality risks and observed proportions of the original model. The sold line (“Bias-corrected”) indicates the agreement between predicted mortality risks and observed proportions of the bootstrap model.

**Fig 3:**
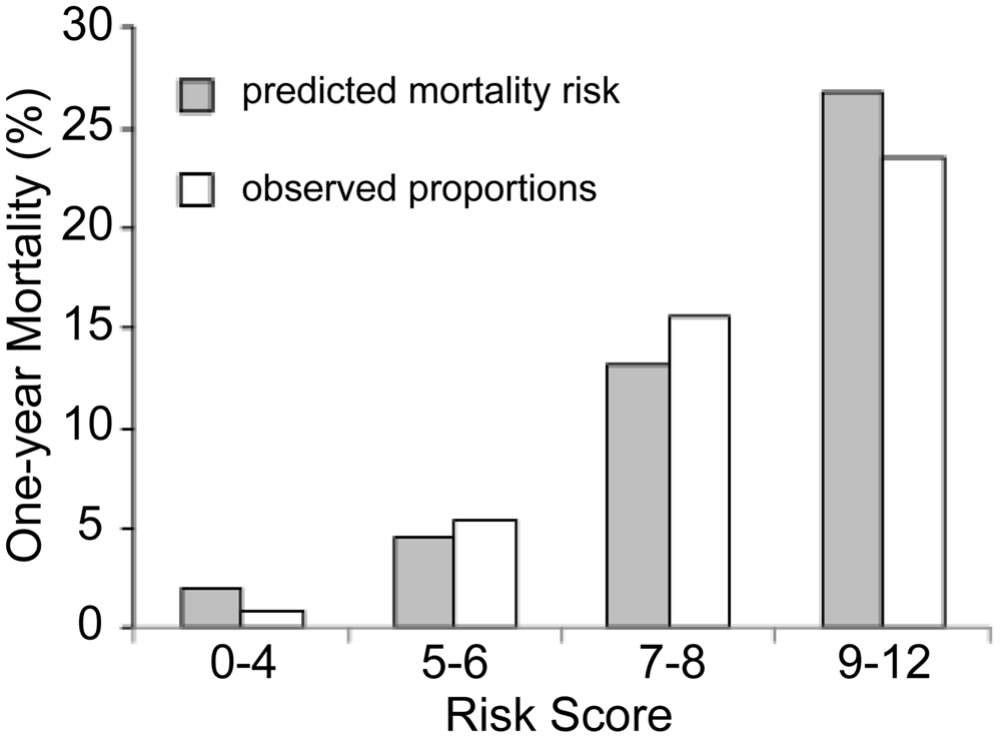
Predicted mortality risks and observed proportions for ranges of total scores. Prognostic score calculated form the following six items well predicts 1-year mortality for patients initiating haemodialysis: high eGFR level (>7 mL/min per 1.73 m^2^), low serum albumin levels, high calcium levels, high modified Charlson Comorbidity Index, low performance status, and no use of ESA. The modified Charlson Comorbidity Index was excluded items related to diabetes and renal disease from the original Charlson Comorbidity Index in the present study.

A majority method was selected to obtain a final set of selected predictors [9]. First, backward elimination was separately applied to each of the five imputed data sets (Fig 1, (2)), resulting in five sets of selected predictors. Second, predictors that were selected in all of the five data sets were chosen as the final set of selected predictors (Fig 1, (3)), with exclusion of some predictors based on balance between number of candidate predictors with number of outcomes (deaths) and discussion according to clinical relevance. Third, the logistic regression with the selected predictors was separately applied to each of the five imputed data sets (Fig 1, (4)). Finally, the mean for each of the five estimates for β-coefficients of the final model were taken and variances of the five estimates were pooled according to Rubin’s rules (Fig 1, (5)) [10, 13].

In a derivation phase, overfitting should be adjusted for, especially when a large number of candidate predictors compared to number of outcomes (deaths) was considered or automated variable selection such as backward elimination was used [14]. To account for this, the β-coefficients derived from each of the imputed data sets were multiplied by a heuristic shrinkage factor, as follows:

$$\left(\chi^{2}{}_{\text{model}}–\;\text{df}\right)/\chi^{2}{}_{\text{model}}$$

where χ^2^_model_ denotes the model chi-square and df denotes the degree of freedom (Fig 1, (5)). This approach is a post hoc method to adjust β-coefficients of the predictors for overfitting and avoids extreme predictions when applied to new patients [11, 15]. The shrunken β-coefficients of the predictors in the final model divided by two-fifths of the two small β-coefficients in the model and rounded up to the nearest integer to give a simple point score (Fig 1, (6)).

### Validation Phase

The validation was performed using single imputed data among the five imputed data generated in the derivation phase, as the single imputation validation is a temporary stand-in for the multiple imputation fit [16]. To validate the final model, we selected a bootstrapping technique with 2000 resamples that, in contrast to the data-splitting technique, preserves the sample size and leads to more precision and power [17]. This technique estimates the likely performance of the prediction rule on a new sample of patients from the same patient population. The ability to discriminate between patients with and without 1-year mortality was assessed by the area under the receiver operating characteristic (ROC) curve. The agreement between predicted 1-year mortality based on the rule and the observed mortality risk was assessed with a calibration plot [15, 17].

Total scores were calculated by adding the scores for each final predictor. Patients were then categorized into total score categories and their observed risks of 1-year mortality were compared.

All statistical analyses were performed using Stata version 11.2 (Stata Corp., College Station, TX, USA) and R software (http://www.R-project.org).

## Results

### Description of Candidate Predictors and Outcome Variable

A total of 688 patients enrolled in this study. The candidate predictors and the outcome variables are described in Table 1. Median age of patients was 69 years, 230 patients (33.4%) were female, and 42.6% (293 of 688 patients) had diabetic nephropathy as an underlying disease. Median HD-free interval from eGFR of 10 mL/min per 1.73 m^2^ was 196 days, median eGFR at initiation of HD was 5.43 mL/min per 1.73 m^2^, and 85.8% (585 of 682 patients) were administered ESA before initiation of HD. Only 11.6% (79 of 684 patients) had a modified CCI of 0 points, and 85.3% (583 of 683 patients) had deteriorated activities of daily living defined as performance status. A total of 62 patients (9.0%) died within one year of starting HD.

### Derivation

Multivariable logistic regression analysis with backward elimination procedure showed that the following variables were retained in all of the five imputed dataset: eGFR>7 mL/min per 1.73 m^2^, serum albumin<3.5 g/dL, calcium>8.5 mg/dL, modified CCI≥1 points (higher risk for ≥3 points), performance status≥1 points (higher risk for 3–4 points), body mass index>18, pulmonary edema, and ESA use (Table 2). Among the eight candidate predictors, two were excluded: body mass index (based on discussion that body height and body weight will be unlikely to be obtained among patients with decreased performance status), pulmonary edema (based on discussion that it was anti-intuitive for future use that odds ratio of presence of pulmonary edema for death was <1.0 in all of the five imputed dataset). Further, based on number of outcomes (62 deaths), combination of the following six predictors were considered to be reasonably fitted to the final multivariable logistic regression model: eGFR>7 mL/min per 1.73 m^2^, serum albumin<3.5 g/dL, calcium>8.5 mg/dL, modified CCI≥1 points (higher risk for ≥3 points), performance status≥1 points (higher risk for 3–4 points), and ESA non-use (Table 3). For the analysis population, calculations showed an area under the ROC curve of 0.829.

**Table 2:** Retained predictors in each of 5 imputed dataset and choice of the predictors.

Predictors	ImputedData 1	ImputedData 2	ImputedData 3	ImputedData 4	ImputedData 5	Choice with further discussion based on clinical relevance
**Age, year**						
**Female gender**						
**Body mass index >18**	X	X	X	X	X	Chosen
**Primary renal disease**	X	X	X		X	
**eGFR>7 mL/min per 1.73 m** ^**2**^	X	X	X	X	X	Chosen
**Urea nitrogen, mg/dL**						
**Haemoglobin, g/dL**						
**Serum albumin<3.5 g/dL**	X	X	X	X	X	Chosen
**Serum potassium, mEq/L**			X		X	
**Serum calcium>8.5 mg/dL**	X	X	X	X	X	Chosen
**Serum phosphorus, mg/dL**						
**C-reactive protein>1.0 mg/dL**		X	X	X	X	
**Modified Charlson Comorbidity Index** ^a^	X	X	X	X	X	Chosen
**Performance status**	X	X	X	X	X	Chosen
**Fatigue**						
**Edema**						
**Pulmonary edema**	X	X	X	X	X	
**Nausea**						
**Dysorexia**						
**Diarrhea**						
**Constipation**				X		
**Other digestive symptom**						
**CNS manifestation**						
**Peripheral nerve abnormalities**						
**Itch**						
**Haemorrhagic diathesis**						
**Hypertension**						
**Diabetic retinopathy**						
**ESA use**	X	X	X	X	X	Chosen

Predictors listed in this table were entered to logistic regression model with backward elimination procedure. X indicates predictors retained after backward elimination procedure in each of the five imputed dataset. Predictors which retained all of the five imputed dataset were considered as candidate predictors of final prediction model. After further discussion based on clinical relevance, six predictors were chosen as the final prediction model.

eGFR, estimated glomerular filtration rate; CNS, central nervous system; ESA, erythropoiesis-stimulating agent

^a^Items related to diabetes and renal disease were excluded from the original Charlson Comorbidity Index in the present study.

**Table 3:** Multivariable predictors of 1-year mortality and associated risk scoring system.

Variables	Adjusted odds ratio	95% confidence interval	β-coefficient^a^	Risk score^b^
**eGFR>7 mL/min per 1.73 m** ^**2**^	2.05	1.13–3.74	0.66	**1**
**Serum albumin<3.5 g/dL**	2.33	0.96–5.63	0.77	**1**
**Serum calcium>8.5 mg/dL**	2.80	1.41–5.59	0.94	**2**
**Modified Charlson Comorbidity Index** ^c^ **(vs. 0)**				
1–2	3.59	1.57–8.20	1.17	**2**
≥3	6.74	2.57–17.6	1.74	**3**
**Performance status (vs. 0)**				
1–2	2.03	0.45–9.13	0.65	**1**
3–4	6.75	1.51–30.1	1.74	**3**
**ESA non-use**	3.29	1.67–6.45	1.09	**2**

eGFR, estimated glomerular filtration rate; ESA, erythropoiesis-stimulating agent

^a^Original β-coefficients multiplied by heuristic shrinkage factor to improve predictions for future patients.

^b^Scores assigned by dividing the shrunken β-coefficients by 0.568 and rounding to nearest integer.

^c^Items related to diabetes and renal disease were excluded from the original Charlson Comorbidity Index in the present study.

### Validation

Predictive discrimination of 1-year mortality was good, with an area under ROC curve of 0.831 (95% confidence interval, 0.789–0.892). The predicted risks of approximately 20% of the apparent model were overestimated, and the predicted risks of >40% were underestimated (Fig 2). The bias-corrected estimate was slightly nonlinear but only slightly improved compared to the apparent calibration. The 90 percentile of absolute error in predicted mortality risks between the bootstrap and original model is 0.013, which suggests only a small degree of bias from over-fitting in the original model.

### Development of Risk Score

Table 4 shows the score chart derived from the final logistic regression model (Table 3) that can be used as a risk score. A median overall risk score of six (range: 0–12) was obtained for each patient in the study population. Fig 3 shows predicted mortality risks and observed proportions for the ranges of total scores. Some discrepancies between predicted risk and observed proportion were observed (Table 5).

**Table 4:** Score chart to predict 1-year mortality risk.

Points	0	1	2	3
**eGFR>7 mL/min per 1.73 m** ^**2**^, **yes**	No	Yes		
**Serum albumin<3.5 g/dL, yes**	No	Yes		
**Serum calcium>8.5 mg/dL, yes**	No		Yes	
**Modified Charlson Comorbidity Index, points** ^a^	0		1–2	≥3
**Performance status, points**	0	1–2		3–4
**ESA non-use, yes**	No		Yes	

Points correspond to each predictor value and are added to give a score.

eGFR, estimated glomerular filtration rate; ESA, erythropoiesis-stimulating agent

^a^Items related to diabetes and renal disease were excluded from the original Charlson Comorbidity Index in the present study.

**Table 5:** Predicted mortality risks and observed proportions for ranges of total scores.

Total Score	Predicted mortality risk	Observed proportion	
		%	(n/N)^a^
0–4	2.5%	1.7%	(4/235)
5–6	5.5%	6.6%	(15/228)
7–8	15.2%	16.6%	(26/157)
9–12	28.9%	25.0%	(17/68)

^a^Number of patients experiencing 1-year mortality/total number of patients in each risk category.

## Discussion

In this multicenter cohort study, we developed a simple risk score using parameters from blood examinations obtained in routine clinical evaluations to predict 1-year mortality in stage 5 CKD patients who were starting HD. This score might provide a personalized assessment of mortality risk of CKD patients under predialysis nephrology care and help nephrologists make a shared decision with patients and their families regarding the initiation of HD.

Our risk score to predict 1-year mortality is composed of the following six items available at initiation of HD: high eGFR level (>7 mL/min per 1.73 m^2^), low serum albumin levels, high calcium levels, high modified CCI, low performance status, and no use of ESA.

These items have a number of clinical implications or similarities to previous studies. First, despite the exclusion of eGFR patients with >10 mL/min per 1.73 m^2^, mortality prediction of high eGFR is in good agreement with a previous study showing that of patients who started HD, those with eGFR of 8–10 mL/min per 1.73 m^2^ had higher mortality than those with eGFR of 4–6 mL/min per 1.73 m^2^ [3]. However, whether the initiation of HD when eGFR levels are high directly causes earlier death is still controversial. Of note, comorbidity, which causes decompensated symptoms related to the renal failure even though eGFR is relatively preserved, might exacerbate poor mortality.

Second, mortality prediction related to high modified CCI, hypoalbuminemia, and low performance status is also compatible with previous reports [5, 18, 19]. Hypoalbuminemia might indicate malnutrition, nephrotic, or chronic inflammatory state and can be partially modified by medical care. Predialysis management for nutrition and proteinuria might therefore improve survival after initiation of HD.

Third, accurate mortality prediction based on elevated serum calcium levels is consistent with previous findings of mortality risk related to abnormally elevated calcium levels among CKD patients and the incidence HD [20–25]. Increased serum calcium levels are generally caused by certain drugs, presence of malignancies or immobility. It might be speculated that one of the reasons of higher serum calcium levels in predialysis CKD patients is to use drugs such as calcium-based phosphate binder, vitamin D or thiazide as it was independent of serum albumin level and lower performance status as proxy for immobility. For example, coronary calcification induced by calcium-based phosphate binders occurred more than that by sevelamer in CKD patients [26].

Fourth, mortality prediction with no use of an ESA before initiation of HD is consistent with previous reports [27, 28]. However, the beneficial effect of an ESA for CKD before the initiation of HD is controversial. In addition, the use of an ESA in the present study might indicate the provision of high quality management by nephrologists, which might be partially supported by higher proportion of the patients receiving an ESA than those in the previous study [29].

Our risk score differs from previous scores with respect to target population and simplicity as well as availability of parameters in a routine nephrology care setting. For example, the prognostic score developed by Couchoud et al. was only applicable to elderly patients [1], whereas our score doesn’t include age. It may be counterintuitive that age was not associated with 1-year mortality. However, it is possible that effect of age on mortality was lessened by adjustment of performance status and comorbidity, as both are associated with aging. Regarding routine nephrology care settings, they included irrelevant patients with unplanned initiation of HD who have higher mortality than those with planned initiation of HD [30], and whether or not their patients received medical care by the nephrologists is unknown. In contrast, our risk score was developed from patients who received predialysis nephrology care and whose eGFR was then 10 mL/min per 1.73 m^2^ in an out-patient setting. Our score might therefore be more accurate than the previous scores for nephrologists when referring to personalized mortality risks to decide whether to initiate HD. The prognostic score developed by van Diepen et al. was also restricted to patients with diabetic nephropathy with a complicated score calculation requiring computer assistance [5]. Chua et al. developed a 5-item prognostic tool with simple integer scores; however, this tool is difficult to use in routine settings due to the requirement of echocardiography measurement [31]. In contrast to these previous studies, our risk score is applicable for CKD patients regardless of age and underlying disease and composed of simple integer scores available in daily nephrology practice settings.

The strength of this study is that the 6-item risk score for predicting 1-year mortality was developed using clinical data available immediately before the initiation of HD from multicenter nephrology institutions. As mortality tends to be relatively high one year after starting HD, this integer risk score might be useful for estimating prognosis, identifying high-risk patients, and making care plans. For example, when bedridden CKD patients with hemiplegia and peripheral vascular disease exhibit a low level of serum albumin and a high level of calcium, predicted 1-year mortality is >25% according to our risk score. The nephrologist would therefore be able to discuss HD initiation based on this predicted mortality with the patient and their family.

However, several limitations also warrant attention in the present study. First, CKD patients who started HD with an eGFR>10 mL/min per 1.73 m^2^ were excluded from this study. This might explain why emergent complications related to CKD, such as pulmonary edema, were not retained in the risk scores. Second, as our risk score was only validated internally, external validation should also be conducted. Third, 1-year mortality in the present population was lower than that reported in the Japanese registry (15.9%) [3]. This lower mortality might be explained by the exclusion of patients with eGFR>10 mL/min per 1.73 m^2^ at initiation of HD, as a higher level of eGFR at the initiation of HD is associated with mortality [4, 18, 19, 32]. In addition, patients enrolled in the present study received predialysis nephrology care at least once, which has been related to improved survival after the initiation of HD [29, 33].

In conclusion, we developed and validated a simple risk score in CKD patients receiving predialysis nephrology care to predict 1-year mortality after the initiation of HD. This score might help nephrologists make personalized assessments for risk of mortality and facilitate making a shared decision with patients and families regarding the initiation of HD.
